# Chagas Disease-induced Sudden Cardiac Arrest

**DOI:** 10.5811/cpcem.2017.5.33626

**Published:** 2017-10-09

**Authors:** Michael M. Neeki, Michelle Park, Karan Sandhu, Kathryn Seiler, Jake Toy, Massoud Rabiei, Sasikanth Adigoupula

**Affiliations:** * Arrowhead Regional Medical Center, Department of Emergency Medicine, Colton, California; † Loma Linda University Medical Center, Department of Cardiology, Advanced Heart Failure and Transplantation, Loma Linda, California; ‡ California University of Science and Medicine, Colton, California

## Abstract

Sudden cardiac death (SCD) is the most common cause of death in patients with Chagas disease (ChD). There are over 300,000 ChD-infected individuals living in the United States, of whom 10–15% have undiagnosed Chagas cardiomyopathy (CCM). CCM patients have a higher risk of SCD compared to non-CCM patients, although early and appropriate treatment of CCM patients can result in a 95% relative risk reduction of SCD. Emergency physicians have a unique opportunity to improve outcomes among these patients by becoming more vigilant in recognizing the signs and symptoms of CCM in patients who present in sudden cardiac arrest. We report the case of a patient presenting to the emergency department with pulseless ventricular tachycardia and an undiagnosed history of CCM.

## INTRODUCTION

Chagas disease (ChD) has become a global public health problem due to high rates of immigration from endemic to non-endemic countries.^1^,^2^ An estimated 7–8 million individuals worldwide are infected with ChD, mostly in Latin America.^3^,^4^ In the United States there are >300,000 ChD-infected individuals, of whom 30,000–45,000 are estimated to have undiagnosed Chagas cardiomyopathy (CCM), the primary form of chronic ChD.^5^,^6^ The Center of Excellence for Chagas Disease reports that about one in five Latin American patients in Los Angeles presenting with non-ischemic cardiomyopathy had CCM.^7^

However, the patient population at risk for ChD infection is not limited to patients of Latin American heritage. While vector-borne *Trypanosoma cruzi* transmission is responsible for most ChD infections, other routes of transmission include blood transfusion, organ transplantation, and vertical transmission from mother-to-fetus.^1^,^3^ Voluntary screening for *T. cruzi* antibodies in U.S. blood donations began in 2007 but only became mandatory in 2016.^8^ Vertical transmission accounts for 63–315 congenitally-infected infants per year in the U.S.^9^ Because these cases are often not recognized or treated, there is an increased risk for these children to develop CCM in adulthood.^5^

There are a growing number of case reports on ChD patients who initially presented with cardiac signs and symptoms that confounded diagnosticians in non-endemic countries (Europe, Japan).^10^–^19^ We describe the case of a patient in the U.S. who presented to the emergency department (ED) with pulseless ventricular tachycardia (VT) and an undiagnosed history of ChD. The goal of this case report is to increase the awareness of ChD among U.S. emergency physicians and, consequently, contribute to reducing the outcome of sudden cardiac death (SCD) in CCM patients.

## CASE REPORT

A 48-year-old Hispanic male presented to the ED complaining of palpitations, shortness of breath, generalized abdominal pain, and lightheadedness for eight hours with worsening symptoms in the preceding two hours. Two months prior to initial presentation, he had experienced these symptoms intermittently and been given recommendations for an outpatient workup; then he was lost to follow-up. Past medical history was significant for hypercholesterolemia and hypertension controlled by enalapril.

On initial presentation, the patient was in moderate respiratory distress but alert and oriented. Examination assessed tachypnea, thready radial pulses and an unobtainable blood pressure. Cardiac telemetry displayed a wide complex VT of 206 beats per minute, as seen in the image below.

As the patient was being prepared for emergent synchronized cardioversion, he began to vomit and became unresponsive and apneic. He remained in VT with no pulse. Advanced cardiac life support was initiated and he received defibrillation at 200 J, biphasic. Epinephrine 1 mg intravenous (IV) bolus was administered every three minutes for a total of three doses. Overall, the patient received six biphasic defibrillation shocks; the first two at 200 J and the last four at 300 J. The patient intermittently regained pulses between shocks and remained hypotensive. After return of spontaneous circulation and normal sinus rhythm, the patient received a bolus of amiodarone 300 mg IV and was started on a drip at 1 mg/min. This was followed by an infusion of dobutamine titrated to 7mcg/kg/min. Levophed was given at 8 mcg/min and later titrated to 2 mcg/min. He was admitted in critical condition to the intensive care unit (ICU). As his initial troponin in the ED was 1.2 ng/mL, the cardiology service was consulted.

Further investigations into his social history uncovered relevant information. The patient’s wife stated that he was a construction worker who was born and raised in southern Mexico and that many people in his village suffered from heart conditions. ChD was considered as a potential diagnosis, and the cardiologist agreed with the primary team to initiate a ChD diagnostic workup.

An echocardiogram displayed severe left ventricular (LV) dysfunction with an ejection fraction (LVEF) of 20%. The following morning, a coronary angiogram demonstrated mild-to-moderate coronary artery disease that was unlikely to have caused his severe cardiomyopathy. Throughout his two-day ICU course, the patient continued to experience recurrent VT but was successfully cardioverted during each event.

Two days later, the laboratory analysis of *T. cruzi* immunoglobulin G (IgG) and immunoglobulin M was found to be remarkable for titers of 1:128 and <1:20, respectively, indicating a past *T. cruzi* infection and confirming chronic ChD. The patient continued to improve throughout his one-week hospital course and was discharged home with referral to the cardiology clinic for implantable cardioverter-defibrillator (ICD) placement.

**CPC-EM Capsule**

What do we already know about this clinical entity?

Chagas disease (ChD) causes Chagas cardiomyopathy (CCM). CCM patients have a higher risk of sudden cardiac death (SCD) than patients with other cardiomyopathies: 60% of CCM deaths are due to SCD.

What makes this presentation of disease reportable?

In the U.S., increasing numbers of patients with undiagnosed chronic ChD are seeking care in the ED for cardiac abnormalities that lead to SCD.

What is the major learning point?

Timely treatment of acute ChD can be curative and prevent CCM. Management of CCM with implantable cardioverter defibrillator placement and amiodarone may reduce the relative risk of SCD by 95%.

How might this improve emergency medicine practice?

Emergency physicians can reduce the risk of SCD among CCM patients by recognizing the risk factors for ChD in patients and by initiating the appropriate management of patients with severe CCM.

## DISCUSSION

The current body of literature reflects an increased awareness among clinicians in certain specialties such as cardiology and infectious diseases of the emergence of ChD in the U.S.^6^,^20^,^21^ However, a 2010 survey administered by the Centers for Disease Control and Prevention (CDC) / Medscape reported that 27–68% (ranged by specialty) of U.S. physicians were not confident in their current knowledge of ChD and that 29–60% of respondents neglected to consider ChD in their differential diagnosis for their patient population.^22^ This lack of familiarity with ChD in the medical community poses significant barriers to accessing appropriate and timely healthcare for ChD patients.^2^,^23^

Delays in the diagnosis of ChD can be devastating to the patient as treatment of an acute infection can cure the patient and prevent chronic complications such as CCM and SCD.^24^,^25^ An acute *T. cruzi* infection consists of a nonspecific febrile illness that resolves within 4–8 weeks, although 5–10% of acutely infected patients rapidly progress to severe myocarditis.^3^,^26^ Most patients remain asymptomatic during a latent (indeterminate) phase that can last 10–30 years.^27^ These patients have the “indeterminate form” of ChD and exhibit normal electrocardiogram (ECG)/echocardiogram findings despite positive serology.^3^,^28^ The remainder of patients develop chronic ChD. Approximately 30% of these chronic ChD patients develop CCM.^25^,^29^ Other less common chronic complications include gastrointestinal megasyndromes (megaesophagus, megacolon) that can exist alone or in conjunction with cardiac disease.^3^,^9^,^15^,^25^

The exact pathogenesis of CCM secondary to chronic ChD is unknown, although it is commonly accepted that persistent parasitic injury to the myocardium causes fibrosis in the posteroinferior/apical left ventricle and sinus node; this fibrosis results in malignant arrhythmias that elicit sudden cardiac arrest and, ultimately, SCD.^26^,^28^,^30^,^31^ CCM is associated with a worse prognosis than non-CCM cardiomyopathies.^7^,^28^,^30^ The World Health Organization estimates that 50,000 deaths per year are related to CCM, mostly due to SCD (60%), heart failure (25%), and thromboembolic events (15%).^28^,^29^,^32^

The severity of adverse outcomes in CCM patients underlines the need for emergency physicians to take a more proactive approach in identifying patients with risk factors for *T. cruzi* exposure. Obtaining a detailed social and medical history is vital to assessing a patient’s risk of *T. cruzi* exposure. Pertinent questions about the patient include birthplace, countries of long-term residence, country of birth mother’s residence, and history of blood transfusions/organ transplantation.^2^ Since 2016, an online calculator has been available to aid clinicians in predicting a patient’s risk of chronic ChD.^2^ Certain characteristics associated with the CCM patient can also help physicians: most are young (30–50 years of age) and present with atypical chest pain, palpitations, bradycardia, syncope, symptoms of congestive heart failure, or thromboembolic manifestations.^25^,^30^,^33^,^34^

The standard 12-lead ECG is the first step in evaluating a patient with suspected ChD and can help emergency physicians to rule-out patients with a low risk of SCD. ChD patients with normal ECGs have prognoses comparable to patients without ChD.^31^ An ECG can also alert physicians to the presence of CCM, even in asymptomatic patients. The most common abnormal ECG finding for severe CCM is right bundle branch block.^9^,^27^ Other common abnormalities include both non-sustained (NSVT) and sustained ventricular tachycardia (SVT), ventricular premature beats, atrio-ventricular block, and prolonged QT intervals.^27^,^31^ ECG abnormalities can be detected several years before the presentation of symptoms or cardiomegaly and can signal the need for a ChD diagnostic workup.^26^,^31^

The diagnosis of ChD requires two or more serologic tests (enzyme-linked immunosorbent assay, immunofluorescence, indirect hemagglutination) to detect *T. cruzi*-specific antibodies or radiographic/echocardiographic evidence of heart disease.^7^,^26^,^28^,^35^ An IgG titer greater than 1:16 is positive for chronic *T. cruzi* infection.^12^

Once the diagnosis is established, risk scores calculating morbidity and mortality for CCM patients can be used to identify patients at higher risk for SCD and other complications. The Rassi score for ChD prognosis employs six factors to predict 10-year all-cause mortality rates: 1) New York Heart Association functional class III or IV; 2) radiological evidence of cardiomegaly; 3) LV systolic dysfunction; 4) NSVT on 24-hour Holter monitoring; 5) low QRS voltage; and 6) male sex.^35^ Patients are classified as low risk with a 10% mortality rate, intermediate risk with a 44% mortality rate, or high risk with an 85% mortality rate.^35^ Our patient had a Rassi score of 18 (high risk) and LVEF<20%, which warranted an ICD placement.^33^

Appropriate management can reduce the occurrence of SCD. SCD accounted for 87% of deaths in CCM patients without pacemakers and 67% of deaths in CCM patients with pacemakers.^32^ ICD implantation is recommended for the prevention of SCD in patients with non-ischemic cardiomyopathy and LVEF≤35% who were resuscitated from sudden cardiac arrest caused by SVT.^27^,^36^ Amiodarone is used as the preferred antiarrhythmic agent in these patients.^7^,^11^,^27^,^28^,^37^ One study reported that treatment of CCM with ICDs and amiodarone resulted in a 95% relative risk reduction in SCD (p=0.006) and a 72% reduction in all-cause mortality (p=0.007).^37^

Other treatment options depend on the stage of ChD. Two antitrypanosomal drugs used to treat acute ChD, benznidazole and nifurtimox, are only available in the U.S. through the CDC.^9^ Treatment is recommended for all pediatric patients and those with reactivation from immunosuppression; due to severe adverse reactions, the regimen is considered on an individual basis for chronic ChD patients and those ≥50 years of age.^10^,^25^,^38^ The effectiveness of either drug for chronic ChD remains controversial.^24^,^25^,^33^,^39^–^41^

Patient education is an essential component of appropriate ChD management as patients are often lost to follow-up. Patients with confirmed ChD infections should be advised against blood or organ donation, while those with suspected ChD should be informed of treatment options for infected women and children to prevent congenital infections and chronic complications.^25^ Emergency physicians can decrease the propagation of acute ChD infection and chronic ChD complications in the U.S. by screening and educating patients at risk for ChD.

## CONCLUSION

Immigration is altering the epidemiology of ChD in the U.S. and increasing the number of ChD-infected patients seeking care in the ED. These changes highlight the need for emergency physicians to update their knowledge of the clinical presentation, diagnostic workup and management of historically neglected diseases to provide patient care that reflects the changing demographic characteristics of their communities.

Our case report endeavors to increase the awareness of the cardiac complications of ChD among U.S. emergency physicians. Emergency physicians have a unique opportunity to reduce the outcome of SCD among CCM patients by becoming more vigilant in recognizing the risk factors for ChD in patients who present with sudden cardiac arrest and by initiating the appropriate management of patients with severe CCM.

## Figures and Tables

**Image:**
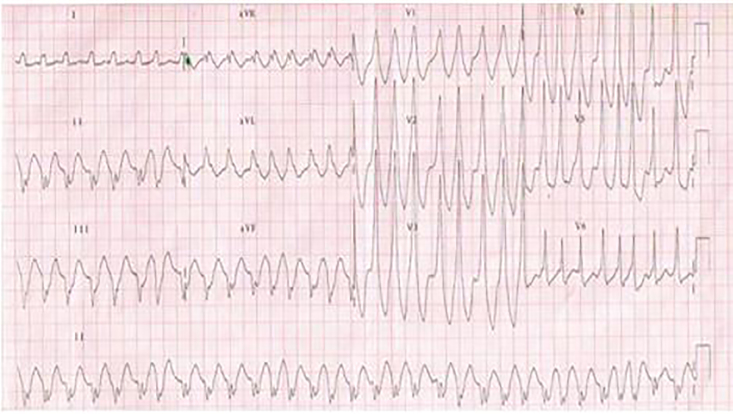
Electrocardiogram displaying ventricular tachycardia at 206 beats per minute.
